# Only Infant *MLL*-Rearranged Leukemia Is Susceptible to an Inhibition of Polo-like Kinase 1 (PLK-1) by Volasertib

**DOI:** 10.3390/ijms252312760

**Published:** 2024-11-27

**Authors:** Jacqueline Fischer, Estelle Erkner, Pia Radszuweit, Thomas Hentrich, Hildegard Keppeler, Fulya Korkmaz, Julia Schulze-Hentrich, Rahel Fitzel, Claudia Lengerke, Dominik Schneidawind, Corina Schneidawind

**Affiliations:** 1 Department of Medicine II, University Hospital Tuebingen, Eberhard Karls University, 72074 Tuebingen, Germany; jacquelinemarie.fischer@med.uni-tuebingen.de (J.F.); pia.radszuweit@med.uni-tuebingen.de (P.R.); dominik.schneidawind@usz.ch (D.S.); 2 Department of Medical Oncology and Hematology, University Hospital Zurich, 8091 Zurich, Switzerland; 3 Department of Genetics/Epigenetics, Faculty NT, Saarland University, 66123 Saarbruecken, Germany; thomas.hentrich@uni-saarland.de (T.H.); julia.schulze-hentrich@uni-saarland.de (J.S.-H.)

**Keywords:** *MLL*-rearranged leukemia, PLK-1, CRISPR/Cas9, volasertib, targeted therapy, cell of origin

## Abstract

*MLL*-rearranged (*MLL*r) leukemia is characterized by a poor prognosis. Depending on the cell of origin, it differs in the aggressiveness and therapy response. For instance, in adults, volasertib blocking Polo-like kinase 1 (PLK-1) exhibited limited success. Otherwise, PLK-1 characterizes an infant *MLL*r signature, indicating potential sensitivity. By using our CRISPR/Cas9 *MLL*r model in CD34+ cells from human cord blood (huCB) and bone marrow (huBM) mimicking the infant and adult patient diseases, we were able to shed light on this phenomenon. The *PLK-1* mRNA level was significantly increased in our huCB compared to the huBM model, which was underpinned by analyzing infant and adult *MLL*r leukemia patients. Importantly, the expression levels correlated with a functional response. Volasertib induced a significant dose-dependent decrease in proliferation and cell cycle arrest, most pronounced in the infant model. Mechanistically, upon volasertib treatment, we uncovered negative feedback only in the huBM model by compensatory upregulation of *PLK-1* and related genes like *AURKA* involved in mitosis. Importantly, the poor response could be overcome by a combinatorial strategy with alisertib, an Aurora kinase A inhibitor. Our study emphasizes the importance of considering the cell of origin in therapeutic decision-making and provides the rationale for evaluating volasertib and alisertib in *MLL*r leukemia.

## 1. Introduction

In recent years, it has become more and more apparent that fundamental differences exist between fetal and adult cells regarding their proliferative behavior, lineage bias, and their response to driver mutations and, consequently, to anti-cancer therapies [1]. Accordingly, the cell of origin where the leukemia initiates plays a critical role in disease pathogenesis. Additionally, the microenvironment and niche in fetal hematopoietic development are crucial for the initiation of *mixed-lineage leukemia* (*MLL*) rearranged infant leukemia [2,3]. Hereby, MLL deficiency led to embryonic lethality and hematopoietic abnormalities, emphasizing its pivotal role in blood cell development [4,5,6]. *MLL*-rearranged (*MLL*r) leukemias thus represent an excellent disease model for investigating the differences in leukemia-initiating cells dependent on the cell of origin. 

The *MLL* gene is commonly involved in leukemia-related chromosomal translocations and is associated with leukemia, particularly high-risk subtypes such as acute lymphoblastic leukemia (ALL) and acute myeloid leukemia (AML) [7]. *MLL* is located on chromosome 11q23 and encodes a histone methyltransferase that regulates gene transcription by modifying histone H3 at lysine 4 (H3K4me) [8,9]. The MLL protein complex controls the transcription of target genes, notably homeobox (*HOX*) genes, which play a crucial role in embryonic development and hematopoiesis [6]. *MLL*-rearranged leukemias are characterized by a wide variety of fusion partner genes, with the eight most common being ALF Transcription Elongation Factor 1 *AFF1* (*AF4*), MLLT3 Super Elongation Complex Subunit *MLLT3* (*AF9*), MLLT1 Super Elongation Complex Subunit *MLLT1* (*ENL*), Histone Lysine Methyltransferase DOT1L Cofactor *MLLT10* (*AF10*), Elongation Factor For RNA Polymerase II *ELL*, Adherens Junction Formation Factor *AFDN* (*AF6*), Epidermal Growth Factor Receptor Pathway Substrate 15 *EPS15*, and partial tandem duplication *PTD*, which are present in over 90% of cases [10]. These translocation partners are typically genes encoding proteins that interact with multimeric biochemical complexes, including nuclear, membrane-associated, or cytoplasmic proteins [7]. It is noteworthy that several common fusion partners, such as *AFF1*, *AFF4*, *MLLT1*, *MLLT3*, and *ELL*, are part of the super elongation complex (SEC), which plays a critical role in transcriptional elongation [11,12]. Upon fusion with *MLL*, the SEC becomes aberrantly stabilized at MLL target genes, including *HOX* genes and the proto-oncogene *MYC*, contributing to the disease’s pathology [11,12,13].

Analysis of *MLL* fusions has shown that the specific translocation is associated with distinct disease phenotypes. *MLL-AFF1* (*MLL-AF4*) is the most frequent translocation in ALL, occurring in 56.5% of cases, while *MLL-MLLT3* (*MLL-AF9*) is more common in AML, present in 30.4% of cases [7]. Moreover, the distribution of *MLL* fusion partners differs between patient age groups. In infants and adults, *MLL-AF4* is most prevalent (40.2% and 48.5%, respectively), whereas in pediatric patients, *MLL-AF9* is more frequent (25.9%) [7]. Significant differences exist between subgroups of *MLL*r leukemias in terms of disease progression, prognosis, and treatment response. Infant *MLL*r leukemias are predominantly associated with B-cell acute lymphoblastic leukemia (B-ALL) and are characterized by a particularly poor prognosis. In contrast, adult *MLL*r leukemias exhibit a myelomonocytic phenotype and are scored with an intermediate prognosis [14,15,16]. However, the presence of *MLL*r in adults with ALL is associated with a poor prognosis and places patients in a high-risk category [17]. 

Given the aggressive nature of *MLL*r leukemias, patients require intensive therapeutic regimens. The standard frontline treatment regimen typically includes cytarabine and anthracyclines (e.g., daunorubicin, idarubicin) to achieve remission. Additional consolidation and maintenance therapy is then provided, depending on the patient’s condition and risk of relapse [17,18]. However, chemotherapy resistance is a common occurrence, and treatment options such as allogeneic hematopoietic stem cell transplantation (HSCT) and CAR T-cell therapy, despite their promising potential, are not without risks of toxicity and adverse effects [19,20]. Consequently, research is focused on the development of targeted therapies, including epigenetic modifications, cell cycle checkpoints, and other molecular interventions, such as those affecting cholesterol metabolism, to improve outcomes for patients with *MLL*-related leukemia [21,22,23]. Therefore, we established a clustered regularly interspaced short palindromic repeats/CRISPR-associated protein 9 (CRISPR/Cas9) induced *MLL-AF9* model system in hematopoietic stem and progenitor cells (HSPCs) derived from both human cord blood (huCB) and human bone marrow (huBM). The use of CRISPR/Cas9 for genetic engineering has significantly improved the efficiency of inducing translocations compared to earlier methods like TALENs [24]. Using this approach, *t(4;11)* and *t(9;11)* translocations were successfully induced in CD34+ HSPCs from huCB as an infant cell model and human bone marrow (huBM) as an adult cell model, leading to the outgrowth of leukemic cells. Single guide RNAs (sgRNAs) targeting common patient-specific breakpoints (*MLL*, *AF4*, *AF9*) were used, allowing the *MLL*r-associated leukemogenesis in vitro [25,26,27]. A specifically composed cytokine mixture sustained cell proliferation and exhibited the translocated cells with a poorly differentiated, myelomonocytic progenitor blast cell phenotype, along with *MLL*r-specific markers (CD32+, CD9+) [21,28]. Our in vitro model is specifically designed to mimic the infant and adult patient diseases, thereby facilitating further insights into disease development and treatment response dependent on the cell of origin [13,28,29,30,31].

Polo-like kinase 1 (PLK-1) belongs to the serine/threonine protein kinases and is known to be critical for cell cycle progression by influencing mitosis entry, centrosome maturation, spindle formation, and separation of chromosomes in the cytokinesis [32,33,34]. One hallmark of malignant transformed clones is the increased proliferative capacity, which makes PLK-1 a promising therapeutic target. Given its importance in malignant diseases, different PLK-1 inhibitors have been developed and already clinically tested for their safety [34,35,36,37,38,39,40,41]. However, although the blocking of PLK-1 was generally well tolerated, the results, especially as monotherapy, were hampered by limited clinical success in adult leukemia patients [42]. Recently, it has been shown that PLK-1 is critical for the survival of *MLL*r ALL cells and is, therefore, potentially a main mechanism in sustaining this disease [43]. By using a variety of pharmaceutical assays, we were able to demonstrate the superior anti-leukemic effect of volasertib in infant in contrast to adult leukemia; we revealed a higher *PLK-1* expression in our infant *MLL*r model and commercially available cell lines (THP-1, SEM). Importantly, our findings correlated with patient samples showing a higher dose-dependent sensitivity towards the inhibition of PLK-1 by volasertib as well. Moreover, volasertib conferred a mitotic arrest by stopping the proliferation and promoting apoptosis. Mechanistically, a transcriptomic analysis of both models upon volasertib inhibition uncovered a compensatory upregulation of *PLK-1* only in the *MLL*r model derived from adult huBM, resulting in poor response to the volasertib treatment. Therefore, the higher expression of *PLK-1* and the absence of this escape mechanism in *MLL*r cells derived from huCB could serve as an explanation for the better response to volasertib in infant *MLL*r leukemia. Mechanistically, the present study demonstrates that combination treatment with alisertib—an Aurora kinase A inhibitor—overcomes the aforementioned lack of treatment response in adult *MLL*r leukemia, possibly by modulating these feedback mechanisms.

Taken together, in our study, we emphasize the importance of considering the different cell of origin in disease development towards a treatment strategy. Our in vitro analysis paves the way for the further evaluation of volasertib and alisertib in *MLL*r leukemia as promising combinatorial therapy in a clinical trial.

## 2. Results

### 2.1. Revealing PLK-1 as a Potential Promising Target in Infant MLL-AF9 Leukemia

**Figure 1:**
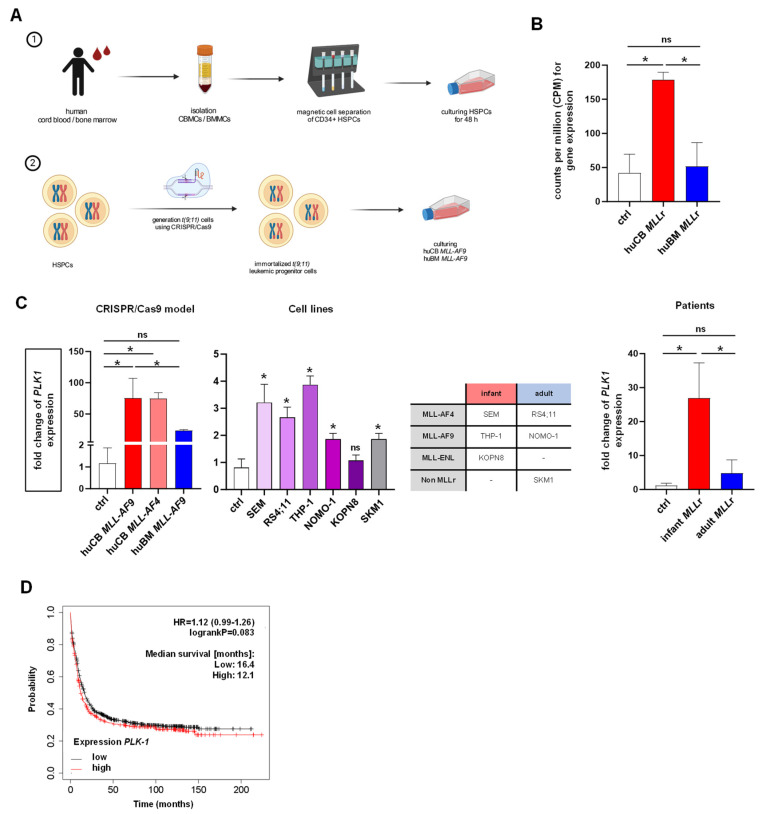
**Revealing PLK-1 as a potential promising target in infant MLL-AF9 leukemia.** (**A**) CD34+ HSPCs were isolated from huCB and huBM via Ficoll separation and magnetic cell separation and cultured for 48 h. Thereafter, *t(9;11)* was induced in cultured HSPCs using the CRISPR/Cas9 system. (**B**) RNA sequencing of human CRISPR/Cas9 *MLL-AF9* cells derived from huCB (*n* = 5) and huBM (*n* = 5) compared with the respective control cells (huCB/huBM-derived CD34+, *n* = 4). Student’s *t*-test. * *p* < 0.05. ns: not significant *p* > 0.05. (**C**) Fold change of *PLK-1* overexpression in huCB *MLL-AF4* and *MLL-AF9* cells (*n* = 5/*n* = 5); huBM *MLL*r cells (*n* = 5); cell lines THP-1, NOMO-1, SEM, RS4;11, KOPN8, and SKM1 (all *n* = 3); and infant and adult *MLL*r leukemia patient samples (*n* = 3/*n* = 3) compared to CD34+ huCB/BM control cells (ctrl, *n* = 4), measured by RT-qPCR. One-way ANOVA. * *p* < 0.05. ns: not significant *p* > 0.05. Overview of *MLL* translocation in the cell lines used. (**D**) Kaplan–Meier survival curve (www.kmplot.com, 26 August 2024). Higher *PLK-1* expression levels in AML patients show a trend to worse survival rates. Logrank. *p* = 0.083. Median survival rates [months]: low *PLK-1* level 16.4, high *PLK-1* level 12.1.

We previously established a CRISPR/Cas9 *MLL-AF9* model, which offers an ideal platform to identify disease mechanisms and potential therapeutic targets in *MLL*r leukemia [28]. For that purpose, we induced *t(9;11) (p22,q23)* by using CRISPR/Cas9 in CD34+ HSPCs derived from both huCB and huBM to mimic infant and adult *MLL*r leukemia, respectively (Figure 1A). In various diseases, it is already known that the cell of origin can be relevant for the resultant immunophenotype and signaling pathways, leading to diverse disease progression and therapeutic responses [44]. Recently, PLK-1 was described as one driver in infant *MLL*r leukemogenesis and maintenance [43], but it is also found in a variety of human cancers with poor prognosis [39]. Likewise, by using transcriptomic analysis with RNA sequencing (RNA-seq), we could also reveal significantly elevated *PLK-1* expression only in the *MLL-AF9* cells derived from huCB and not from huBM (Figure 1B). Importantly, to confirm our RNA-seq results, we used RT-qPCR, showing comparable results: huCB *MLL-AF4* and *MLL-AF9* cells express *PLK-1* significantly higher compared to huBM *MLL-AF9* cells and CD34+ huBM/CB control cells (Figure 1C). To further support our findings in differential expression levels of *PLK-1* dependent on the cell of origin, we assessed *PLK-1* expression in commercially available *MLL*-rearranged cell lines, with THP-1 as the infant and NOMO-1 as the adult *MLL-AF9* cell line and SEM as the infant and RS4;11 as the adult *MLL-AF4* cell line, as well as KOPN8 (*MLL-ENL*) and SKM1 (non *MLL*r) cell lines. Importantly, we were able to show the most pronounced overexpression in the infant *MLL-AF4* and *MLL-AF9* leukemic cell lines (Figure 1C). Our findings are also supported by analysis of *PLK-1* expression in *MLL*r cells derived from infant and adult patients (Figure 1C). In addition, analysis of publicly available data from acute myeloid leukemia (AML) patients showed a trend towards decreased overall survival in adult leukemia cases with higher *PLK-1* expression, which extended beyond those with *MLL* fusions (Figure 1D). Unfortunately, there were no data on childhood leukemia available, but this highlights the oncogenic and pivotal role of PLK-1 in leukemogenesis and as a potential target. In summary, our findings suggest that the PLK-1 pathway may be of particular significance, especially in the context of infant *MLL*r leukemia.

### 2.2. MLLr Leukemia Cells Derived from huCB Are More Susceptible to PLK-1 Inhibition Than Those from huBM 

**Figure 2:**
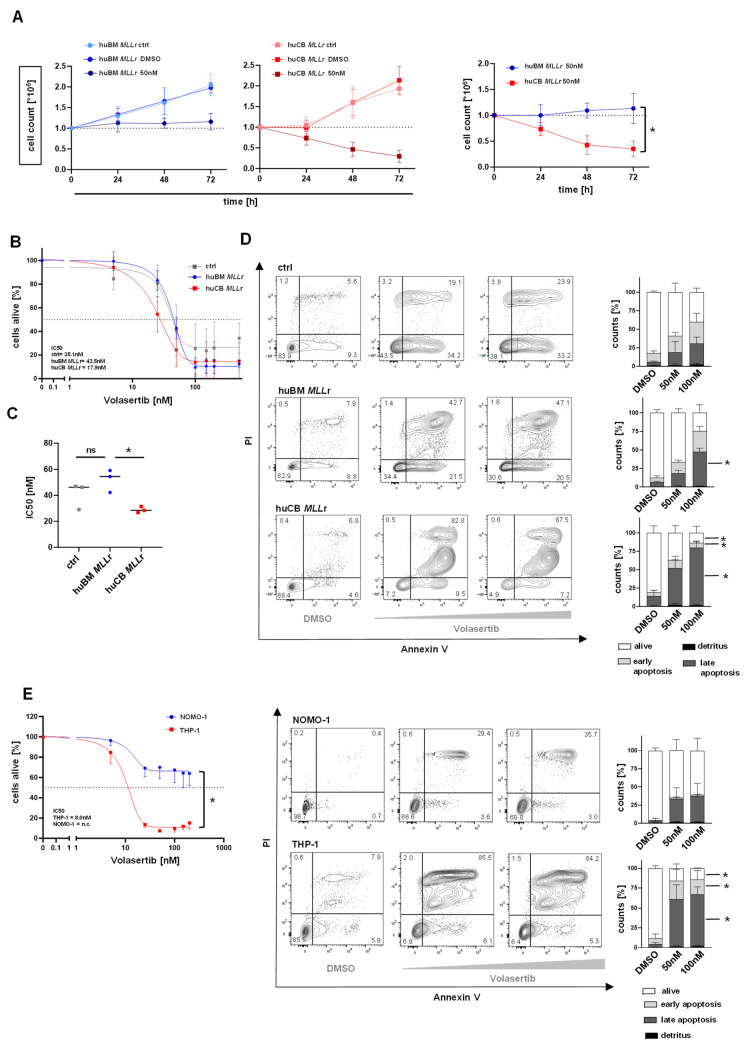
***MLL*r leukemia cells derived from huCB are more susceptible to a PLK-1 inhibition than those from huBM.** (**A**) Cell counts were assessed following treatment with volasertib (50 nM), vehicle control (DMSO), or no treatment (as a baseline control) in huCB and huBM CRISPR/Cas9 *MLL*r cells (*n* = 3 each). Relative cell counts were determined using a Neubauer counting chamber after Trypan blue staining and normalized to the vehicle control (DMSO). Right: Significant difference of proliferation between huCB and huBM CRISPR/Cas9 *MLL*r cells at 72 h after 50 nM volasertib treatment. One-way ANOVA. * *p* < 0.05. (**B**) huCB and huBM CRISPR/Cas9 *MLL*r (*n* = 3/*n* = 3) and CD34+ huCB/BM control cells (ctrl, *n* = 4) were treated with increasing concentrations of volasertib or vehicle control (DMSO) for 72 h. Relative cell count was determined by counting cells in a Neubauer counting chamber after Trypan blue staining, normalized to vehicle control (DMSO). IC50 values: huBM *MLL*r 43.5 nM, huCM *MLL*r 17.9 nM, ctrl 35.1 nM. IC50 values of the dose-dependent curves were interpolated from a four-parameter logistic model. (**C**) Significant difference between IC50 values of huBM *MLL*r and huCB *MLL*r cells compared to ctrl and each other. One-way ANOVA. * *p* < 0.05. ns: not significant *p* > 0.05. (**D**) Representative flow cytometric histograms of Annexin V/PI staining to determine the apoptotic effect of 72 h volasertib treatment (DMSO, 50 nM, 100 nM) on huCB and huBM CRISPR/Cas9 *MLL*r (*n* = 3/*n* = 3) and CD34+ huCB/BM control cells (ctrl, *n* = 4), measured by flow cytometry. On the right, summarized fractions normalized to their own vehicle control (DMSO). One-way ANOVA. * *p* < 0.05. (**E**) Left: THP-1 and NOMO-1 (*n* = 3/*n* = 3) cells were treated with increasing concentrations of volasertib or vehicle control (DMSO) for 72 h. Relative cell count was determined by counting cells in a Neubauer counting chamber after Trypan blue staining and normalized to vehicle control (DMSO). IC50 values: THP-1 8.0 nM; NOMO-1 could not be determined, as 50% cell death was not achieved (n.c.). IC50 values of the dose-dependent curves were interpolated from a four-parameter logistic model. Significant difference between endpoints (200 nM volasertib treatment) of NOMO-1 and THP-1. One-way ANOVA. * *p* < 0.05. Right: Representative flow cytometric histograms and summarized distribution of Annexin V/PI staining to determine the apoptotic effect of volasertib treatment (DMSO, 50 nM, 100 nM, 72 h incubation) on NOMO-1 and THP-1 (*n* = 3/*n* = 3) cells measured by flow cytometry. On the right, summarized fractions normalized to their own vehicle control (DMSO). One-way ANOVA. * *p* < 0.05.

To further evaluate the functional relevance of the observed differences in *PLK-1* expression in our infant and adult CRISPR/Cas9 *MLL-AF9* models as a potential target, we used the PLK-1-inhibitor volasertib (BI6727) in pharmaceutical assays. To identify the optimal treatment window, we used our CRISPR/Cas9-engineered huCB/huBM *MLL*r models to assess the effects of volasertib on cytotoxicity and cell proliferation over time. Compared to the vehicle control (DMSO) and untreated cells, a 72 h treatment was found to be the most effective, significantly reducing cell numbers in *MLL*r cells derived from huCB, while *MLL*r cells derived from huBM remained mainly unaffected and cell proliferation stagnated. (Figure 2A). Next, we treated our CRISPR/Cas9-generated huCB/huBM *MLL*r cells and control cells with increasing volasertib concentrations for 72 h and assessed the dose-dependent inhibition of cell proliferation by Trypan blue staining and microscopy. Accordingly, we were able to generate the respective dose–response profiles (Figure 2B). Interestingly, cell proliferation of huBM-derived *MLL*r cells was similar to the CD34+ control cells only mildly affected by volasertib treatment, whereas the proliferation of huCB-derived *MLL*r cells was significantly reduced. Correspondingly, the IC50 values of huCB-derived *MLL*r cells were significantly lower compared to the values observed in control or huBM-derived *MLL*r cells (Figure 2C). Next, we performed Annexin V staining and flow cytometry to evaluate the induced apoptosis following 72 h treatment with different volasertib concentrations (Figure 2D). By using 100 nM volasertib, over 95% of cells of the *MLL*r cells derived from huCB were dead or in late apoptosis (Annexin+, PI+/−) (Figure 2D). By contrast, using the same volasertib concentration, significantly more CD34+ control cells (40% viable cells) and *MLL*r cells derived from huBM (30% viable cells) were alive (Annexin-, PI-) (Figure 2D). We, therefore, assume that volasertib has a specific anti-leukemic effect on infant *MLL*r cells by inducing apoptosis and cell death. To further confirm these findings, we also treated the infant *MLL*r cell line THP-1 and the adult *MLL*r cell line NOMO-1 with increasing volasertib concentrations for 72 h and, accordingly, analyzed cell proliferation and cell viability by Annexin staining. Likewise, volasertib mainly affects the infant *MLL*r cell line, whereas both proliferation and apoptosis in the adult *MLL*r cell line stagnates and leaves 60% of the cells alive (Figure 2E). We found that this effect also applies to *MLL-AF4* cell lines, with the infant cell line SEM showing greater sensitivity to volasertib treatment compared to the adult cell line RS4;11 (Figure S1A). In addition, we can show that the infant *MLL-ENL* cell line KOPN8, as well as the non-*MLL*r cell line SKM1, are unaffected by a volasertib treatment (Figure S1A). Our findings demonstrate that volasertib has a stronger impact on infant *MLL*-*AF4* and *MLL-AF9* leukemia cells in contrast to adult *MLL*r or non-*MLL*r leukemia, suggesting a novel and promising treatment approach for this disease.

### 2.3. Inhibition of PLK-1 Leads to Reduced Viability and Mitotic Arrest

**Figure 3:**
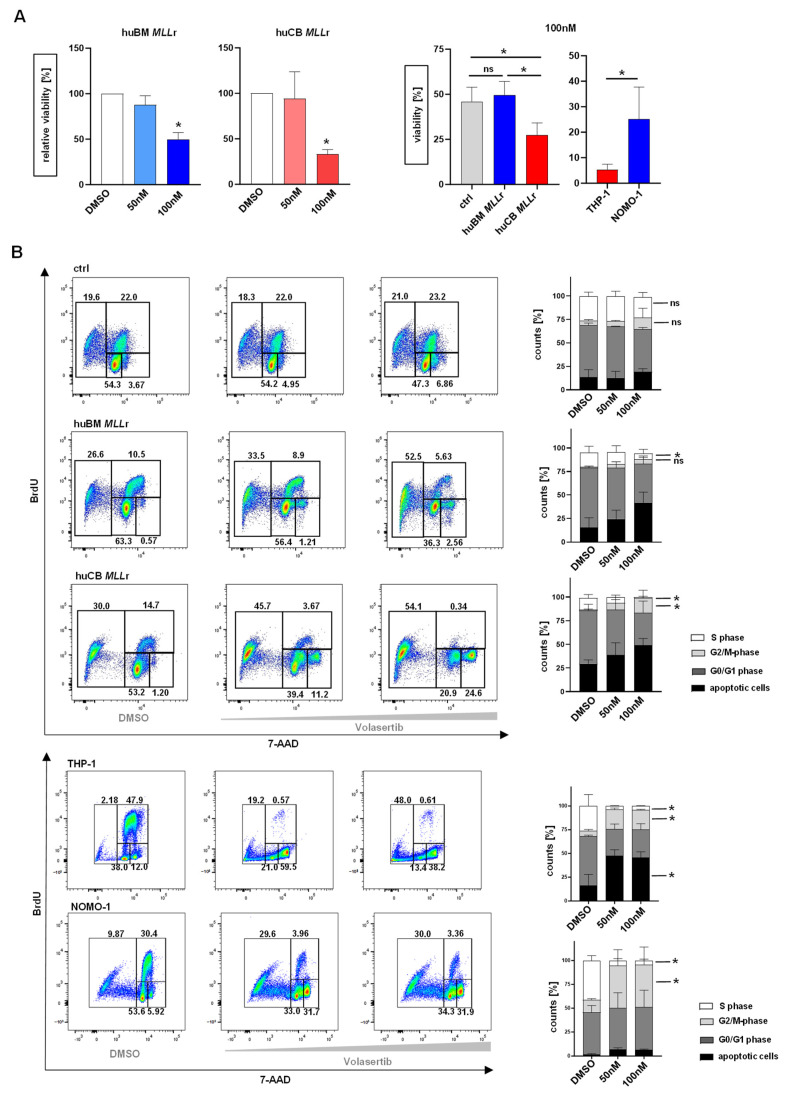

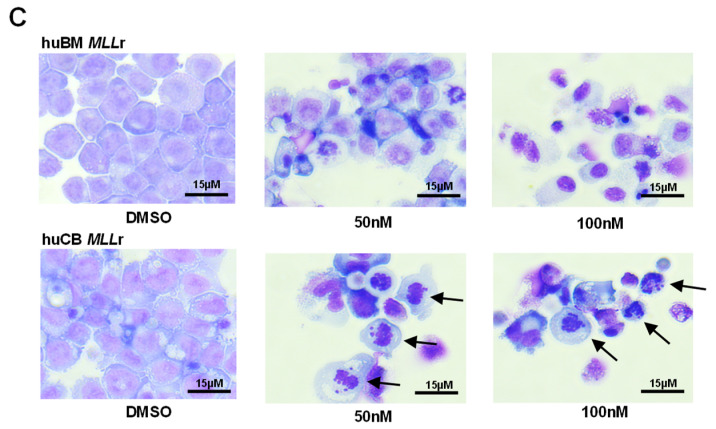
**Inhibition of PLK-1 leads to reduced viability and mitotic arrest.** (**A**) The 72 h volasertib treatment (DMSO vehicle control, 50 nM, 100 nM) on huBM and huCB CRISPR/Cas9 *MLL*r cells (*n* = 3/*n* = 3) decreased cell viability, measured by AlamarBlue viability assay. Comparison of the reduction in cell viability after treatment with 100 nM volasertib between huBM and huCB CRISPR/Cas9 *MLL*r cells (*n* = 3/*n* = 3) and CD34+ huCB/BM control cells (ctrl, *n* = 4). One-way ANOVA. * *p* < 0.05. ns: not significant *p* > 0.05. (**B**) Representative (left) and pooled (right) data of BrdU cell cycle analysis of huBM and huCB CRISPR/Cas9 *MLL*r cells (*n* = 3/*n* = 3), CD34+ huCB control cells (ctrl, *n* = 3), and THP-1, NOMO-1 (*n* = 3/*n* = 3) after volasertib treatment for 48 h (DMSO vehicle control, 50 nM, 100 nM). This shows a significant increase in G2/M-phase and a decrease in S-phase in CB *MLL*r cells. Normalized to respective vehicle control (DMSO). One-way ANOVA. * *p* < 0.05. ns: not significant *p* > 0.05. (**C**) Images show representative morphologies of huBM and huCB CRISPR/Cas9 *MLL*r cells after volasertib treatment (DMSO vehicle control, 50 nM, 100 nM). Black arrows point at mitotic figures; cells arrested in M-phase. Pappenheim staining.

Besides the impact of the PLK-1 inhibition by volasertib on cell proliferation and apoptosis, we further investigated the impact on cell viability. Therefore, we treated our CRISPR/Cas9-generated huCB/huBM *MLL*r, CD34+ huCB/BM control (ctrl) cells and the cell lines NOMO-1, THP-1, SEM, and RS4;11 for 72 h with the PLK-1 inhibitor volasertib or vehicle control (DMSO). Using the AlamarBlue viability assay, we revealed a dose-dependent and significantly reduced cellular viability in both the infant and adult *MLL*r models and cell lines compared to their own vehicle control (DMSO) (Figure 3A and Figure S1C). However, compared to the control cells, we assessed only a significant reduction in cell viability after 100 nM volasertib treatment regarding the infant *MLL*r cells, which was not observed in the adult *MLL*r cells (Figure 3A). As PLK-1 is known as a key regulator of mitotic progression [39], we further investigated the impact of PLK-1 inhibition by volasertib on the cell cycle. For that purpose, we performed bromodeoxyuridine (BrdU) and 7-amino-actinomycin D (7-AAD) staining and flow cytometry after 72 h volasertib treatment. As expected, we revealed a significant reduction in the S-phase and a significant increase in the M-phase only in the infant *MLL*r cells upon treatment (Figure 3B). Identical treatment in *MLL*r cell lines showed a significant reduction in the S-phase and an increase in the M-phase; however, only THP-1 cells show a significant increase in the apoptotic cell fraction (Figure 3B and Figure S1B). It is known that PLK-1 inhibition results in mitotic arrest [45], specifically a cessation of cell division during mitosis, which is shown through the rise in the M-phase in Figure 3B (7-AAD+/BrdU+) in the *MLL*r cells derived from huCB (Figure 3B). We did not observe an increase in the M-phase in either the *MLL*r cells derived from huBM or the control cells (Figure 3B). To underpin our observation, we performed May–Gruenwald–Giemsa staining on CRISPR/Cas9 *MLL*r cells derived from huCB or huBM after volasertib or DMSO treatment to evaluate alterations in their morphology. Importantly, both CRISPR/Cas9-generated *MLL*r cell lines were characterized by a blastic morphology with a huge nucleus, which is partially differently shaped and contains multiple nucleoli, as well as a basophilic plasma, consistent with patient leukemic cells (Figure 3C). Following inhibitor treatment, we observed an increase in cells with mitotic figures as a sign of mitotic arrest and multiple apoptotic cells with pyknotic nuclei in the infant *MLL*r cells. In contrast, adult *MLL*r cells show fewer mitotic figures and more viable cells after the same dose of volasertib treatment, demonstrating the lower effect of volasertib on adult *MLL*r leukemia cells. These data suggest that the inhibition of PLK-1 results in convincing anti-leukemic effects by the reduction in viability, induction of cell cycle arrest, and, finally, apoptosis in infant *MLL* fusion protein-driven leukemia with significantly less impact on both adult *MLL*r leukemia and control cells.

### 2.4. Transcriptomic Analysis Revealed Compensatory PLK-1 Feedback Mechanism Only in Adult MLLr Cells upon Volasertib Treatment

**Figure 4:**
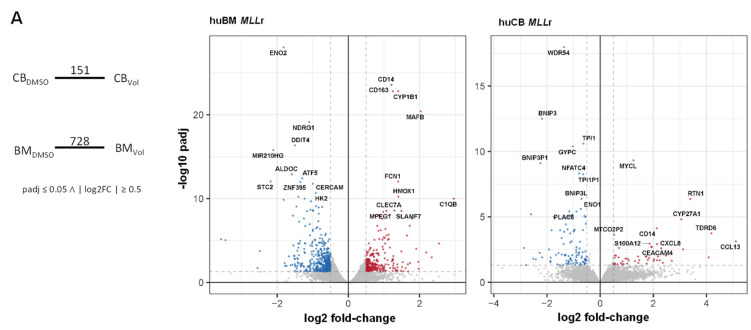

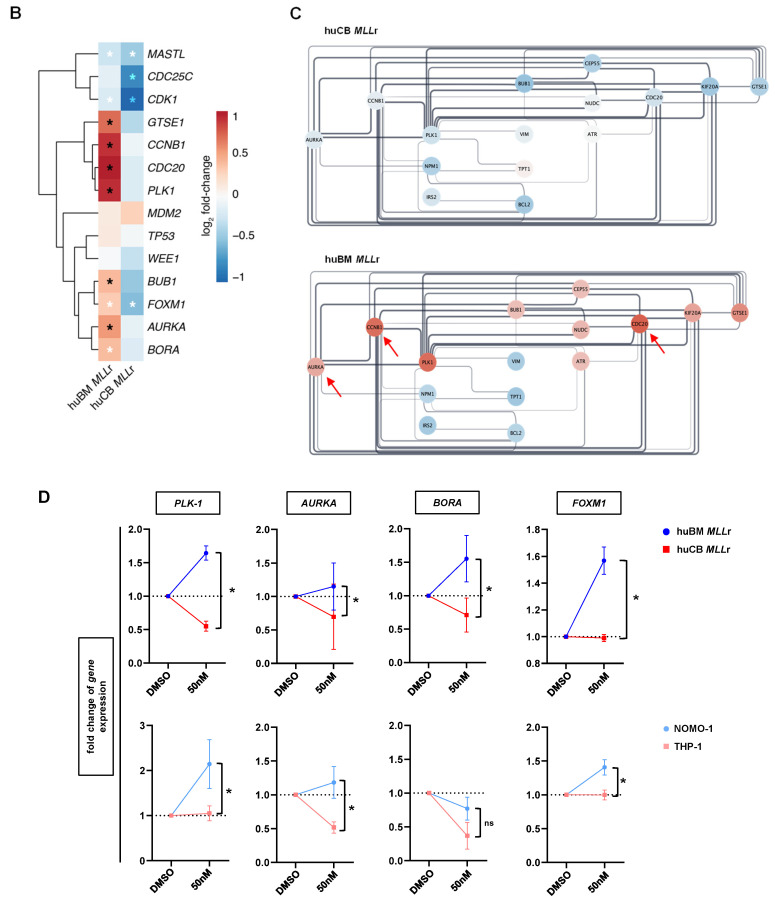
**Transcriptomic analysis** revealed a compensatory PLK-1 feedback mechanism only in adult *MLL*r cells upon volasertib treatment. huBM and huCB CRISPR/Cas9 *MLL*r cells (*n* = 4/*n* = 3) were treated with 50 nM volasertib or vehicle control (DMSO) for 72 h and used for RNA-seq. (**A**) Analysis revealed in huBM *MLL*r cells 728 differentially expressed genes (DEGs) and 151 DEGs in huCB *MLL*r cells after volasertib treatment. Volcano plot of huBM and huCB CRISPR/Cas9 *MLL*r cells after volasertib treatment highlighting downregulated (blue) and upregulated (red) DEGs. Dotted lines indicate significant thresholds (pFDR ≤ 0.05, |log2(fold-change)| ≥  0.5). (**B**) log2(fold-change) of DEGs are shown for *PLK-1* and associated genes, normalized to own vehicle control (DMSO). Black * p_adjust value, white * p_nominal value. huBM *MLL*r cells show an upregulation of PLK-1-related gene pattern, whereas the pattern of huCB *MLL*r is downregulated. (**C**) Interactome of significant altered normalized Reads per Kilobase Millions (nRPKMs) around *PLK-1* in huBM and huCB CRISPR/Cas9 *MLL*r cells reveals potential feedback mechanism. Upregulation (red), downregulation (blue). Additional RNA-seq analysis regarding phosphorylation activity and targets around *PLK-1* are highlighted with red arrows. (**D**) Fold change of *PLK-1*, *BORA*, *AURKA,* and *FOXM1* in huBM and huCB CRISPR/Cas9 *MLL*r cells (*n* = 4/*n* = 3), as well as NOMO-1 and THP-1 cells (*n* = 3/*n* = 3), after 72 h 50 nM volasertib treatment compared to vehicle control (DMSO) measured by RT-qPCR. One-way ANOVA. * *p* < 0.05.

To shed light on the potential mechanisms behind the different treatment responses between infant and adult *MLL*r leukemia cells, we performed RNA-seq after 72 h volasertib (50 nM, 100 nM) or DMSO treatment and compared the gene expression profiles. In *MLL*r cells derived from huCB, we found 151 differentiated genes, whereas *MLL*r cells derived from huBM showed 728 differentially expressed genes (DEGs) (Figure 4A). We listed the top 100 significant up- and downregulated genes in adult (Table S1) and infant *MLL*r cells (Table S2), respectively, and showed all DEGs as a volcano plot in Figure 4A. The most up- and downregulated genes were highlighted in red or blue, respectively. Within these DEGs in both diseases, we found genes involved in myeloid differentiation (CD14, CD163, *Ficolin-1* (*FCN1*), *Macrophage Expressed 1* (*MPEG1*)), whereas glucose metabolism (*Enolase 2* (*ENO2*), *Stanniocalcin 2* (*STC2*)), as one hallmark of leukemogenesis [46], was decreased upon treatment. Despite some similarities in both models, we were curious about variations in their expression patterns depending on the volasertib treatment, potentially explaining the superior sensitivity in our infant model. Therefore, we specifically focused on *PLK-1* and *PLK-1*-associated genes [32,47,48,49,50,51,52,53,54,55,56,57,58,59] (Figure 4B). Surprisingly, we discovered opposite effects in these *PLK-1*-related downstream signaling pathways (Figure 4B). Upon inhibition with volasertib, as expected, *PLK-1* and its target genes were significantly downregulated in infant *MLL*r cells. In contrast, in adult *MLL*r cells, we found an upregulation of *PLK-1* and its target genes. To provide a further description of the downstream signaling pathways involved in the avoidance of the anti-leukemic effect of volasertib treatment in adult but not infant *MLL*r leukemia, we have illustrated these genes in an interactome, with *PLK-1* as the central regulator of the mitosis machinery (Figure 4C). In our adult model, we observed a reinforcement of G2 and S-phase-expressed 1 (*GTSE1*) [56] or Cell Division Cycle 20 (*CDC20*) [60] as a mitotic player of progression. Likewise, other mitotic kinases, like Aurora kinase A/B (*AURKA*, *AURKB*) and Mitotic Checkpoint Serine/Threonine-Protein Kinase BUB1 (*BUB1*), as well as NudC Domaining Containing 1 (*NUDC1*) and Kinesin family member 20A (*KIF20A*) as substrate and transporter of chromosomes involved in the mitotic function of PLK-1, were consequently upregulated [48]. To explore the potential underlying upstream mechanism of the rebound phenomenon of PLK-1 upon inhibition, leading to this re-increased mitotic progression in adult *MLL*r cells, we discovered CyclinB1 (*CCNB1*) as potentially responsible for the observed phenomenon. *CCNB1* is known to be important for mitosis initiation and regulation [61] and hereby positively influences the activity of PLK-1 by the phosphorylation of Aurora kinase A Activator (BORA), an important co-factor of Aurora kinase A (AURKA), which, in turn, activates PLK-1 [57] (Figure 4C). As PLK-1 is involved in the phosphorylation of many proteins, we analyzed our RNA-seq data regarding phosphorylation targets and could confirm an overlap between these results and the interactome (Figure 4C, highlighted genes with red arrows). Especially genes like *AURKA* or *CCNB1* seem to play an important role in kinase activity around *PLK-1*, which underlies our presumption of relevant feedback mechanism. Additionally, we verified our RNA-seq data by performing qPCR of the most important genes, which have attracted attention in our network analysis (Figure 4D and Figure S2). There, we can show the upregulated transcription factor Forkhead Box M1 (*FOXM1*), which intensifies the transcription of CyclinB1, as well as PLK-1, retroactively, resulting in positive reinforcement of PLK-1, and could, therefore, serve as an escape from the toxic treatment effect of volasertib in adult *MLL*r cells [48,49,51,54,55,56,57,58,59,62,63,64,65,66,67,68]. To validate our findings, we also treated the THP-1 (infant) and NOMO-1 (adult) cell lines with volasertib and assessed these relevant pathway genes. Similarly, we observed an increase in the adult cell line NOMO-1, whereas the infant *MLL*r cell line THP-1 showed a decrease in gene expression for *PLK-1*, *AURKA*, and *FOXM-1* (Figure 4D). Our findings indicate that a compensatory upregulation of PLK-1 upon inhibition, leading to the reinforcement of the mitotic machinery, is responsible for the relative insensitivity of the adult *MLL*r cells towards volasertib treatment. Mechanistically, reactivation of the upstream-situated CyclinB1 and FOXM1 could lead to the rebound increase in PLK-1.

### 2.5. Combinational Treatment with Volasertib and the Aurora Kinase a Inhibitor Alisertib Shows Synergistic Effects in the huBM MLLr Model

**Figure 5:**
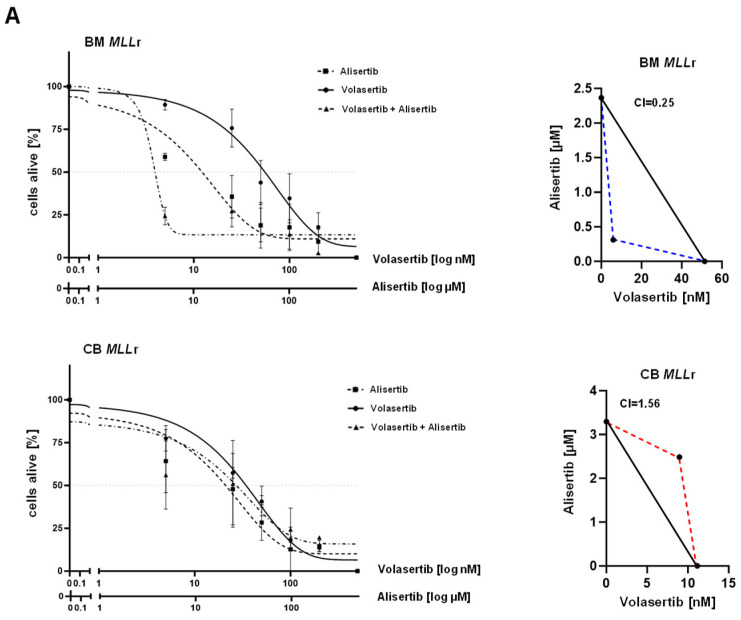

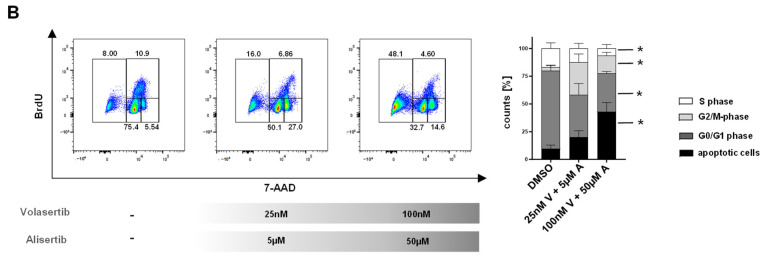
**Combined treatment of volasertib and alisertib in *MLL*r cells.** (**A**) huCB *MLL*r and huBM *MLL*r (*n* = 3/*n* = 3) cells were treated for 72 h with increasing concentrations of volasertib alone, alisertib alone, or in combination. The percentage of viable cells (Annexin V-, PI-) was determined by flow cytometry. In the isobologram, IC50 values were mapped, and the Chou–Talalay method was used to measure the CI for the identification of synergistic effects. CI: huBM *MLL*r 0.25, huCB *MLL*r 1.52. (**B**) Representative (**left**) and pooled (**right**) data of BrdU cell cycle analysis of huBM *MLL*r cells (*n* = 3) after combinatorial treatment with volasertib and alisertib for 72 h (DMSO vehicle control, 25 nM V + 5 µM A, 100 nM V + 50 µM A). This shows a significant increase in G2/M-phase and apoptotic cells and a decrease in S-phase. Normalized to respective vehicle control (DMSO). One-way ANOVA. * *p* < 0.05.

As presented in Figure 4D, huBM *MLL*r cells show an increased gene expression level of *PLK-1* and *AURKA* after volasertib treatment, indicating a potential feedback mechanism. To examine this phenomenon and to overcome the poor response rate towards a volasertib treatment in huBM *MLL*r cells, we investigated a combinational treatment with volasertib and with the Aurora kinase A inhibitor alisertib. Therefore, we treated the huBM and huCB *MLL*r cells with alisertib and volasertib alone or in combination. After 72 h, the number of viable cells was determined by Annexin V staining (Annexin-, PI). The dose–response curves for the inhibitors were then interpolated from the data using a four-parameter logarithmic model (Figure 5A). The IC₅₀ values were plotted in isobolograms, and the corresponding combination index (CI) was calculated according to the Chou–Talalay method (Figure 5A) [69,70] Interestingly, the combination of volasertib and alisertib demonstrated a synergistic effect in huBM *MLL*r cells (CI 0.25), while no advantage was observed in huCB *MLL*r cells treated with the combinatorial approach (CI 1.56). To validate the mechanistic background of both inhibitors, we performed BrdU cell cycle staining and demonstrated that in huBM *MLL*r cells, the combination treatment resulted in a notable reduction in the S- and G0/G1-phase and a significant increase in the M-phase and cell death (Figure 5B). These data suggest that the feedback mechanism of volasertib in adult *MLL*r cells is related to an upregulation of *AURKA* that can be effectively counteracted with a combinatorial treatment approach by using volasertib and alisertib.

## 3. Discussion

*MLL*r leukemia is initiated by a translocation of the *MLL* gene to over 100 known fusion partners in hematopoietic stem and progenitor cells (HSPCs), thereby generating a highly oncogenic fusion protein that drives the disease with fewer secondary mutations than other leukemias [7,71]. Despite the genetic simplicity of *MLL*r leukemia, it has been proven to be difficult to develop faithful in vivo disease models [72]. Especially with respect to the different cell of origin where the mutation occurs, which plays a pivotal role in disease pathogenesis and results in specific behavior towards treatment approaches, even less is known [73]. In this study, we used our innovative CRISPR/Cas9 *MLL*r model with indefinite growth potential in in vitro culture systems, based on patient-specific complete translocations of the *MLL* and *AF9* gene in human HSPCs derived from huCB and huBM, hereby mimicking exactly the infant and adult diseases. Our models allow us, in comparative analysis, to unravel differences dependent on developmental origin. Strikingly, we identified PLK-1 as a driver of infant but not adult *MLL*r leukemogenesis, suggesting it as a potential promising targeted therapy approach in this subtype of *MLL*r leukemia. PLK-1 is one of the conserved mitotic kinases that regulates a number of cellular processes, including mitotic entry, bipolar spindle formation, and kinetochore–microtubule attachment [74]. Therefore, PLK-1 acts as a master regulator of cell division and vulnerability, especially in cancer cells, leading to an ideal target to interrupt the dysregulated proliferative rates in malignancies. Thus, the frequently observed overexpression of *PLK-1* and its oncogenic role in several cancers has led to the development of PLK-1-specific inhibitors, which have already been tested in dose escalation trials for adult acute myeloid leukemia, chronic myeloid leukemia, myelodysplastic syndrome, and in children with various solid tumors, but to our knowledge, not in infant leukemia so far [42]. By using RNA-seq and RT-qPCR, we revealed *PLK-1* was specifically upregulated in *MLL*r leukemia derived from infant cells in comparison to healthy but also adult leukemic cells. These results were supported by analysis with commercially available infant and adult cell lines and, moreover, with primary patient material in comparison, consequently showing a more pronounced *PLK-1* expression in the infant-derived leukemia cells. Our study shows that blocking the PLK-1 signaling pathway by using the PLK-1 inhibitor volasertib is a particularly effective treatment strategy in infant *MLL*r leukemia. By treating huCB-derived *MLL*r cells and the infant-derived leukemia cell lines like THP-1, we were able to show the anti-leukemic effects of volasertib by observing significant changes in cell proliferation, cell viability, and apoptosis upon volasertib treatment in a dose-dependent manner. In contrast, *MLL*r cells derived from huBM or commercially available cell lines, like NOMO-1 derived from an adult donor and the non-*MLL*r cell line SKM-1, showed more resistance to the drug, potentially explaining the observed modest results in clinical trials.

Concerning the relative insensitivity of cell killing behind volasertib in adult *MLL*r cells, we could demonstrate a compensatory upregulation of PLK-1 upon inhibition after 1 day of treatment. This is also supported by our observation in our dose-dependent experiments using volasertib in increasing concentrations with infant and adult *MLL*r cells in comparison, where the adult cells ceased proliferation and apoptosis despite increasing concentrations, indicating upcoming resistance of these cells. To shed light on the potential mechanistic changes upon treatment in both tissues, we performed RNA-seq and revealed that both models shared significantly upregulated genes involved in glucose metabolism and myeloid differentiation. Most importantly, we also found opposite effects, especially in regard to the *PLK-1* expression and the related PLK-1 genes involved in this pathway, leading to a reactivation of the mitotic machinery solely in adult *MLL*r cells. Interestingly, we were also able to uncover an upstream upregulated mechanism that might be responsible for the reactivation loop of PLK-1: CyclinD1, Aurora kinase A, and BORA synergistically phosphorylate PLK-1, leading to an activation of PLK-1 and reopening mitotic entry [49,55]. Additionally, we found that elevated levels of FOXM1 as a transcription factor of PLK-1 support this feedback loop in a mutual activation of both [32,68].

Besides the problem of the potential cell origin-specific behavior of volasertib as an explanation of the hampered success in the previous clinical trials in leukemic adults, another problem describes the usage of PLK-1 inhibitors as monotherapy. Due to the heterogenicity and resistant potential of hematological diseases, combinatory treatment strategies should always be favored. Therefore, other trials have already combined PLK-1 inhibitors with either low-dose cytarabine or hypomethylating agents like decitabine in patients with relapsed/refractory AML or ineligible for intensive chemotherapy, resulting in an increase in the anti-leukemic potential [75]. However, although initially the combinatorial treatment seemed to improve the objective response rate and overall survival, there was also an increased risk of severe infections, limiting the success [36]. Therefore, other combinatorial strategies, especially targeted therapies that could potentially have fewer side effects and cause less damage to the immune system, could be more beneficial. As observed in our transcriptomic analysis, *CCNB1* was elevated in the adult model and was potentially responsible for the reactivation of PLK1. Moreover, elevated levels of *CCNB1* and *PLK-1* are known to be strongly associated with low overall survival in other solid tumors like breast cancer, demonstrating their potentiality as prognostic markers and as combinatorial drug targets [53]. However, one of the top candidates responsible for *PLK-*1 re-upregulation in adult leukemia cells upon volasertib treatment was Aurora kinase A. A number of Aurora kinase A inhibitors have already shown convincing results, for example, alisertib, which is currently undergoing clinical evaluation [76]. By using a combinatorial approach with alisertib, we were able to significantly improve the modest effect of volasertib as a monotherapy, as evidenced by synergistic effects. Importantly, alisertib had no additional beneficial effect in the infant *MLL*r model, where we had not previously observed AURKA upregulation following volasertib treatment. Other groups have also demonstrated a synergistic effect of simultaneously blocking AURKA and PLK-1 in preliminary studies in diffuse midline glioma [77], paving the way for this promising combinatorial strategy to be translated into clinical trials. Therefore, our study not only uncovers the relevance of PLK-1 in leukemogenesis in a tissue-specific manner but also provides a convincing basis to further escalate the therapeutic targeting of PLK-1 and combine treatment strategies in our model and clinical studies in the future to combat master oncogenic drivers in *MLL*r leukemia.

## 4. Materials and Methods

### 4.1. Human CRISPR/Cas9-MLLr Model

Human bone marrow (huBM) from adult donors was obtained from the Department of Hematology and Oncology of the University Hospital Tuebingen (IRB approval 309/2018BO2). Human umbilical cord blood (huCB) was donated by the Center for Women’s Health (Department of Gynecology) of the University Hospital Tuebingen (IRB approvals 751/2015BO2 and 461/2022BO2). Written consent was obtained from all patients in compliance with the Declaration of Helsinki. CD34+ HSPCs were isolated from huCB and huBM by Ficoll separation and MACS according to the manufacturer’s instructions (Miltenyi Biotech, Bergisch Gladbach, Germany). CRISPR/Cas9 was used to induce *MLL-AF9* and *MLL-AF4* translocation, as previously described [28]. Therefore, 1 µg of Cas9 protein (PNAbio, Newbury Park, CA, USA) was incubated with 1 µg of the corresponding transcribed sgRNA (MLL, AF4, AF9, all designed and generated as previously described [28]) for 15 min at room temperature (RT). Then, 3 × 10^5^ HSPCs per reaction were centrifuged (300× *g*, 5 min, RT) and resuspended in 17 µL P3 solution (Lonza Group AG, Basel, Switzerland). Electroporation was performed using the 4D-NucleofectorTM X Unit (Lonza Group AG, Basel, Switzerland); cells were incubated for 3 min and the reaction stopped with stem cell medium SCM: StemMACS HSC Expansion Media XF (Miltenyi Biotec, Bergisch Gladbach, Germany) supplemented with 10% heat-inactivated fetal bovine serum (FBS) (Gibco, Grand Island, NE, USA); 1% Penicillin/Streptomycin, 10,000 U/mL (Lonza, Basel, Switzerland); 50 ng/mL G-CSF (Granocyte, Chugai Pharmaceutical Co., Tokio, Japan); FLT3-L, IL-3, IL-6, SCF, and TPO (PeproTech, Cranbury, NJ, USA); and 0.75 µM SR-1 and UM-729 (Stemcell Technologies, Vancouver, BC, Canada). *MLL*r cells were plated in 24-well plates for long-term cultivation with a cell density of 0.75 × 10^6^ cells/mL and cultured in SCM at 37 °C and 5% CO_2_.

### 4.2. Cell Lines

NOMO-1 (DSMZ ACC 542), KOPN8 (DSMZ ACC 552), and RS4;11 (DSMZ ACC 508) cells were cultured in Roswell Park Memorial (RPMI) 1620 medium (Thermo Fisher Scientific, Waltham, MA, USA) with 10% heat-inactivated FBS and 1% Penicillin/Streptomycin, 10,000 U/mL. THP-1 (DSMZ ACC 16) and SKM1 (DSMZ ACC 547) cells were cultured in Roswell Park Memorial (RPMI) 1620 medium (Thermo Fisher Scientific, Waltham, MA, USA) with 10% heat-inactivated FBS and 1% Penicillin/Streptomycin, 10,000 U/mL. SEM (DSMZ ACC 546) cells were cultured in Iscove’s Modified Dulbecco’s Medium (IMDM) (Thermo Fisher Scientific, Waltham, MA, USA) with 10% heat-inactivated FBS and 1% Penicillin/Streptomycin, 10,000 U/mL.

### 4.3. Quantitative Reverse Transcriptase-PCR (RT-qPCR)

RNA was isolated using the NucleoSpin RNA Kit according to the manufacturer’s instructions (Macherey-Nagel, Dueren, Germany). For cDNA synthesis, 1 µg of RNA was diluted with RNase-free water in a total volume of 11.5 µL and preheated with Random Hexamers at 65 °C for 5 min. Per vial, 7.5 µL Master Mix (Table S3, all Thermo Fisher Scientific, Waltham, MA, USA) was added according to the manufacturer’s protocol. cDNA was synthesized using an appropriate program (Table S4). RT-qPCR for *PLK-1*, *AURKA*, *BORA*, *FOXM1*, *CDC20*, and *GSTE1* (Table 1 Primer Sequences, all by Sigma-Aldrich, St. Louis, MO, USA) was performed using a Maxima SYBR Green qPCR Master Mix (Thermo Fisher Scientific, Waltham, MA, USA) according to the manufacturer’s protocol (Table S5). A Maxima Probe qPCR Master Mix (Thermo Fisher Scientific, Waltham, MA, USA) was used for the amplification of the housekeeper *18S* rRNA (Table 1, Sigma-Aldrich, St. Louis, MO, USA) (Table S6). Analysis was performed with a LightCycler 480 Instrument II (Roche Life Science, Penzberg, Germany) (Table S7). The fold change of gene expression was calculated according to the 2^−ΔΔCT^ method and normalized to *18S* rRNA in relation to the respective control cells.

### 4.4. Inhibitor Treatment Assay

Volasertib BI6727 (Selleck Chemicals LLC, Houston, TX, USA) was diluted in DMSO at the used concentrations. *MLL-AF9* cells and CD34+ control cells derived from both huCB and huBM were seeded with 7.5 × 10^5^ cells/mL. Volasertib was subjected to the cells or cell lines and incubated for 48/72 h at 37 °C in the respective medium described above.

Alisertib MLN8237 (Selleck Chemicals LLC, Houston, TX, USA) was diluted in DMSO at the used concentrations. *MLL-AF9* cells derived from both huCB and huBM were seeded with 7.5 × 10^5^ cells/mL. Alisertib was subjected to the cells or cell lines and incubated for 48/72 h at 37 °C in the respective medium described above.

For combinatorial treatment, alisertib and volasertib were diluted in DMSO at the used concentrations. *MLL-AF9* cells from both huCB and huBM were seeded with 7.5 × 10^5^ cells/mL. Volasertib and alisertib were subjected to the cells and incubated for 48/72 h at 37 °C in the respective medium described above.

### 4.5. Microscopy-Based Determination of Cell Counts

The total viable cell number was counted with 0.04% Trypan blue (Sigma-Aldrich by Merck KGaA, Darmstadt, Germany) at a ratio of 1:10 using a Neubauer counting chamber (Karl Hecht GmbH & Co., KG, Sondheim von der Rhön, Germany). Cell number was calculated by the following formula:

$$\frac{cells}{ml}=\frac{living\;cells}{number\;of\;counted\;squares}\times dilution\;facor\times 10^{4}$$

### 4.6. Cell Viability Assay

To determine cell viability, 10 µL AlamarBlue Cell Viability Reagent (Invitrogen, Waltham, MA, USA) was subjected to 90 µL of cell suspension and incubated at 37 °C for 2 h according to the manufacturer’s protocol. The metabolized fluorochrome was detected on a Tecan Infinite M Plex Microplate Reader (Tecan, Maennedorf, Switzerland) at 560 nm.

### 4.7. Cell Cycle and Apoptosis Analysis

The Annexin V apoptosis assay and BrdU cell cycle analysis were performed using the FITC Annexin V Apoptosis Detection Kit I (BD Biosciences, Franklin Lakes, NJ, USA) and the FITC BrdU flow kit (BD Biosciences, Franklin Lakes, NJ, USA) according to the manufacturer’s protocol.

### 4.8. May-Gruenwald-Giemsa Staining

Cytospins were prepared by centrifuging 100 µL of cell suspension (4 min; 700 rpm; 21 °C) with a Shandon Cytospin 3 centrifuge (Thermo Fisher Scientific, Waltham, MA, USA) and stained with May–Gruenwald–Giemsa dye as previously described [78]: May–Gruenwald solution 5 min, rinse in distilled water, Giemsa solution 10 min, rinse in distilled water. Images were taken using a Zeiss Primovert with a ×40 objective and an Axiocam 105 color camera using ZEN software Version 3.0 blue edition (all Carl Zeiss AG, Oberkochen, Germany, https://www.zeiss.com/microscopy/de/produkte/software/zeiss-zen.html, accessed on 11 February 2019) at a resolution of 2560 × 1920 pixels.

### 4.9. Statistical Analysis

Statistical analysis was performed using one-way ANOVA or student’s *t*-test as indicated in each figure legend. *p*-values < 0.05 were considered statistically significant. IC50 values of the dose–response curves were interpolated from a four-parameter logistic model as previously described [28,69]. All data were analyzed with Prism 7.03 (GraphPad Software, La Jolla, CA, USA).

### 4.10. RNA Sequencing

RNA was isolated (Machery Nagel NucleoSpin RNA Kit, Dueren, Germany) and quality was assessed with NanoDrop (Thermo Fisher Scientific Inc., Waltham, MA, USA) and Bioanalyser measurements (Agilent, Santa Clara, CA, USA). Sequencing libraries were constructed with NEBNext Ultra II directional RNA library kit for Illumina according to the manufacturer’s instructions. Raw sequencing data were processed with the nf-core/rnaseq [79] pipeline v3.10.1 using Star-Salmon against the GRCh38 human genome assembly and v109 of the Ensembl gene annotations. Expression values were imported into R and processed with DESeq2 v1.44.0 [80]. Low-expressed genes (<20 mean normalized reads across samples per tissue) were removed, leaving 14.714 and 16.980 genes for bone marrow and cord blood, respectively. For determining differential expression with respect to treatment, the donor was corrected for. Genes were deemed differential when they had a |log2 fold-change| > 0.5 and a Benjamini–Hochberg adjusted *p*-value < 0.05. nRPKMs (normalized reads per kilobase per million total reads) were calculated from raw counts from DESeq2 [81]. Networks of differential genes were constructed based on String [82] 12.0, enriched with curated interactions of IntAct [83] v247, and visualized in Cytoscape [84].

All raw data underlying this study were deposited in NCBI’s Gene Expression Omnibus (GEO) and are accessible through accession number GSE276639.

## Figures and Tables

**Table 1:** Primer sequences used for RT-qPCR.

Gene	Forward Primer Sequence 5′-3′	Reverse Primer Sequence 5′-3′
***PLK1***	GCCCCTCACAGTCCTCAATAAAG	TCTCTCGAACCACTGGTTCTTCTT
***AURKA***	TGGTCGCCCTCTGGGTAAAGGA	TCCAAGTGGTGCATATTCCAGA
***BORA***	AACAAACTCTCGCCAGTCCT	GACGATGAATATCTTCTGGGTCTA
***FOXM1***	TCCAACATCCAGTGGCTTCG	TCATGCGCTTCCTCTCAGTG
***CDC20***	CGCTATATCCCCCATCGCAG	AGCCGAAGGATCTTGGCTTC
***GTSE1***	CGGGATGTTCTCCCTGACAA	AGGAGGACTTCCTTGCGAGA
***18S***	CGGCTACCACATCCAAGGAA	GCTGGAATTACCGCGGCT

## Data Availability

For the original data, please contact corina.schneidawind@usz.ch.

## Supplementary Materials

The following supporting information can be downloaded at: https://www.mdpi.com/article/10.3390/ijms252312760/s1.
