# Supplementary material for: Only Infant MLL-Rearranged Leukemia Is Susceptible to an Inhibition of Polo-like Kinase 1 (PLK-1) by Volasertib
Source: Int J Mol Sci. 2024 Nov 27;25(23):12760. doi: 10.3390/ijms252312760 (PMC11641557; doi:10.3390/ijms252312760)
Supplement: Supplementary file 1 [file ijms-25-12760-s001.zip › ijms-3260536-supplementary.pdf]

Supplementary materials

Supplementary Figures

Supplementary Figure S1

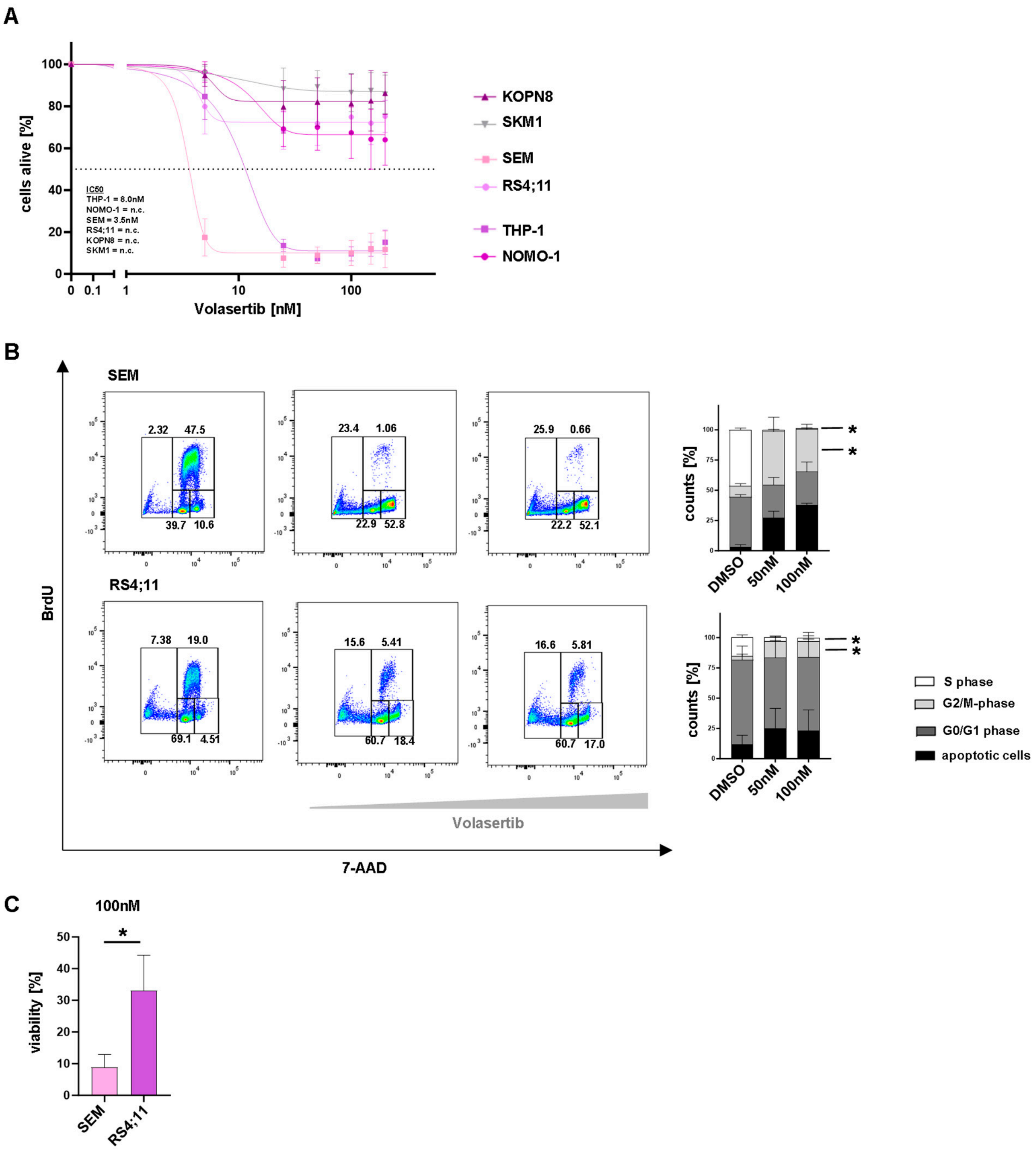

Supp. Figure S1: Volasertib treatment on *MLL*r leukemia cell lines (A) THP-1, NOMO-1, SEM, RS4;11, KOPN8 and SKM1 cells (all n=3) were treated with increasing concentrations of volasertib or vehicle control (DMSO) for 72 h. Relative cell count was determined by counting cells in Neubauer counting chamber after Trypan blue staining, normalized to vehicle control (DMSO). IC50 values: THP-1 8.0 nM, SEM 3.5 nM. NOMO-1, RS4;11, KOPN8 and SKM1 value could not be calculated (n.c.) because the endpoint was not reached. IC50 values of the dose-dependent curves were interpolated from a four-parameter logistic model. **(B)** Representative (left) and pooled (right) data of BrdU cell cycle analysis of SEM and RS4;11 cells (n=3/n=3) after volasertib treatment for 48 h (DMSO vehicle control, 50 nM, 100 nM).

Showing significant increase of G2/M-phase and decrease of S-phase. Normalized to respective vehicle control (DMSO). One-way ANOVA. *p<0,05. ns: not significant p>0.05. **(C)** 72 h volasertib treatment on SEM and RS4;11 cells (n=3/n=3) decreased cell viability, measured by AlamarBlue viability assay. Comparison of the reduction in cell viability after treatment with 100 nM volasertib between SEM and RS4;11 cells normalized to own vehicle control (DMSO). One-way ANOVA. *p<0,05.

Supplementary Figure S2

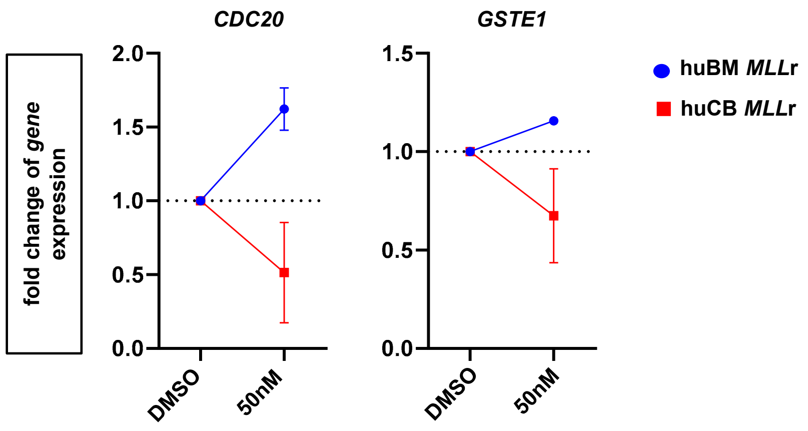

Supp. Figure S2. Elevated gene expression level of huBM *MLL*r cells after volasertib treatment. Fold change of *CDC20* and *GTSE1* in huBM and huCB CRISPR/Cas9 *MLL*r cells (n=3/n=3) after 72 h volasertib treatment compared to vehicle control (DMSO), measured by RT-qPCR.

Supplementary Tables

Supplementary Table S1. Most differentially expressed genes of huBM *MLL*r cells treated with volasertib compared to DMSO control. huBM *MLL*r cells were treated with vehicle control (DMSO) or 50 nM volasertib for 72 h. Four biological replicates (n=4) were used for transcriptomic analysis (RNA-Seq). Top 100 up- and downregulated genes compared to vehicle control DMSO, ranked by adjusted p-value.

Gene	log_2FoldChange	adj. p-value
ENO2	-1,81732386	9,1216E-29
CD14	1,21240516	2,6643E-24
CYP1B1	1,40301108	1,552E-23
CD163	1,25096542	1,552E-23
MAFB	2,03321059	4,0234E-21
NDRG1	-1,10170478	7,4611E-20
DDIT4	-1,50262574	4,5432E-17
MIR210HG	-2,10773805	1,6052E-16
ALDOC	-1,5857519	1,2382E-13
ATF5	-1,29662812	3,7364E-13
STC2	-2,18484383	8,8724E-13
FCN1	1,39971502	9,3581E-13
ZNF395	-1,35207301	1,0502E-12
CERCAM	-0,99996538	1,6885E-12
HK2	-0,91396359	2,0379E-11
HMOX1	1,40459388	5,8257E-11
GBE1	-1,40099285	6,2286E-11
C1QB	2,96457664	1,008E-10
HILPDA	-1,2282352	1,008E-10
PPFIA4	-1,81740743	1,3729E-10
PFKFB4	-1,05003714	2,0748E-10
AK4	-0,90788537	7,8207E-10
P4HA2	-1,4588419	8,6956E-10
RNASET2	-0,73551209	9,8777E-10
VLDLR	-0,83418572	1,1599E-09
MYLIP	-1,06456452	2,2304E-09
GGT5	-0,99344163	2,376E-09
CLEC7A	1,29511088	2,6587E-09
SLAMF7	1,49114641	2,9109E-09
BNIP3L	-0,99294985	2,9109E-09
MPEG1	1,06951277	2,9109E-09
PDK1	-1,15844075	3,1309E-09
CYBB	0,98064064	3,7254E-09
CDT1	-0,83269063	6,4094E-09

HDC	1,17087024	8,5202E-09
CD300E	0,84701403	9,2784E-09
TYMP	0,75283099	1,0568E-08
HAL	-1,55787702	1,0768E-08
CCNB1	0,89951177	1,2967E-08
PLA2G7	1,78811853	2,3882E-08
RASGEF1A	-1,24883866	2,3882E-08
FPR1	0,95756179	2,4814E-08
SLC2A3	-0,84039045	5,0317E-08
MT2A	-1,38175094	5,5238E-08
BPI	-1,08671599	1,1225E-07
CCL2	1,72541339	1,7518E-07
NPL	0,81320419	1,8479E-07
DPYSL3	-1,20928151	1,884E-07
CDHR1	-0,87352489	2,5902E-07
CCDC26	-0,89850816	3,3675E-07
RRAGD	-0,79524847	3,9649E-07
ARHGAP31	1,04272501	4,1464E-07
ASS1	-0,99074721	4,9237E-07
PLK1	0,91273837	5,795E-07
HSPA8	0,84671379	7,0095E-07
MXI1	-0,99896687	7,0095E-07
ERO1A	-0,63924324	1,6159E-06
LILRB2	1,13196203	1,9983E-06
ZNF292	-0,76271035	2,0836E-06
VEGFA	-0,64559857	2,4306E-06
LPAR4	-1,07615937	2,4306E-06
HSPH1	1,07278943	2,4931E-06
GIMAP4	1,64410256	2,5361E-06
CDC20	0,99358888	4,2508E-06
AQP9	1,0252673	4,7219E-06
SLC2A14	-0,79610484	5,1311E-06
STARD4	-0,72892122	6,2714E-06
MIR22HG	1,1374648	6,5443E-06
JAML	0,87217192	6,6268E-06
LEP	-3,57401009	6,8021E-06
ITGAL	-0,53782377	7,1913E-06
WDR54	-0,93846752	7,5697E-06
SLC27A4	0,95950611	7,7318E-06
TP53I3	1,11370318	7,9513E-06
SAMHD1	0,73499502	8,138E-06
NUPR1	-3,45049152	8,9529E-06
PLEKHH3	-0,93223739	9,1764E-06
RPL12P4	-0,71136312	1,0884E-05
SPAG4	-1,35443167	1,1316E-05

COL23A1	-0,5676412	1,2741E-05
SPON2	-1,00285013	1,3114E-05
COL24A1	-0,67438723	1,6187E-05
BSPRY	-0,82273346	1,7157E-05
CTSG	-0,75978068	1,7251E-05
PPP1R3B	-0,74836704	1,8304E-05
CCND2	-0,80843118	1,9416E-05
RGS16	-1,22234228	2,1733E-05
LINC00926	-1,01992548	2,1733E-05
MTCO2P12	0,57798536	2,1961E-05
CITED4	-0,63395867	2,2065E-05
DARS1	-0,66760441	2,2065E-05
EFEMP2	-1,20730847	2,2065E-05
SLC43A2	1,03243382	2,2546E-05
SH3D21	-1,40811443	2,2629E-05
NRP2	2,55030321	2,2629E-05
ZC3HAV1L	-1,37305321	2,2629E-05
TENT5A	-0,70364395	2,438E-05
LINC00899	-0,90668643	3,448E-05
ARMH1	-0,67069844	3,8141E-05
MYBPH	-1,54426279	3,8141E-05

Supplementary Table S2. Most differentially expressed genes of huCB *MLL*r cells treated with volasertib compared to DMSO control. huCB *MLL*r cells were treated with vehicle control (DMSO) or 50 nM volasertib for 72 h. Three biological replicates (n=3) were used for transcriptomic analysis (RNA-Seq). Top 100 up- and downregulated genes compared to vehicle control DMSO, ranked by adjusted p-value.

Gene	log_2FoldChange	adj. p-value
WDR54	-1,35669803	1,0811E-18
BNIP3	-2,18733892	3,1381E-13
TPI1	-0,62892086	2,5093E-11
GYPC	-1,0275518	4,1263E-11
MYCL	1,25358855	4,7962E-10
BNIP3P1	-2,2451882	7,8939E-10
NFATC4	-0,79073876	4,756E-09
TPI1P1	-0,63538403	5,491E-09
BNIP3L	-0,69953145	4,1507E-07
RTN1	3,39114866	4,3213E-07
ENO1	-0,7281724	2,4558E-06
PLAC8	-1,15852535	3,8653E-06
GPI	-0,89125868	3,8653E-06
MIR210HG	-2,6030955	6,3613E-06
PACSIN3	-1,04926189	6,8383E-06
FAM162A	-0,59297586	8,5232E-06
LDHA	-1,14089327	8,5232E-06
GAPDHP1	-0,54854365	1,0222E-05
LDHAP5	-1,2019548	1,0222E-05
ALDOC	-1,74543682	1,0222E-05
CYP27A1	3,05341553	1,5367E-05
GAPDH	-0,53399135	3,3924E-05
PFKFB4	-1,2855422	3,6834E-05
CD14	2,12923224	7,3536E-05
P4HA1	-1,22477712	9,0911E-05
OLMALINC	-0,96385576	9,6339E-05
TPI1P3	-0,6495609	0,00010104
BEND5	-1,82680058	0,00010495
GBE1	-0,97503359	0,00010495
ADD2	-1,50328617	0,00010507
MXI1	-1,00051555	0,00013318
SAP30-DT	-1,35182383	0,0001377
TDRD6	4,18518983	0,00018041
LDHAP7	-1,12488922	0,00018248
MTCO2P2	0,52769181	0,00023843
AK4	-1,44503745	0,00040621
ANKRD37	-1,0758604	0,00072924
CCL13	5,10500167	0,00074002

ECHDC3	-0,75813503	0,00079748
S100A12	1,86081333	0,00111676
HILPDA	-1,37263991	0,00117053
CXCL8	2,13894481	0,00120978
CEACAM4	1,92629354	0,00189259
CD163	1,93793235	0,00205827
RGS16	-0,8899255	0,00207314
ADRA2C	-2,86584825	0,00237954
NCEH1	0,71135843	0,00238454
PGK1	-0,77676348	0,00252475
CXCL3	2,29944937	0,00254452
TREM2	3,12209186	0,00301247
ENO2	-1,92370151	0,00307094
HK2	-0,90094676	0,0032583
NRM	-0,55748179	0,00442321
SCD	-0,65599416	0,00505155
CBX2	-0,89981092	0,00505155
CXCR4	-0,78079689	0,00525355
PKLR	-2,27384746	0,00560496
EFEMP2	-0,81118933	0,00560496
ERFE	-1,56685794	0,00572474
LY6E	-0,37850551	0,00691586
STARD4-AS1	-1,44684953	0,00735488
PPFIA4	-1,66193976	0,0079551
NREP	-1,29870091	0,0079551
MTCO2P12	0,5501096	0,00854462
KLHL23	-0,8819904	0,00854462
AK4P3	-1,33349041	0,00854462
AK4P1	-1,31914649	0,00895259
CCL2	2,47833945	0,00895259
SEMA7A	0,59563763	0,00919611
PLCXD1	-1,18568247	0,00979631
OLFML2B	1,7401029	0,00997755
ZNF141	-0,50888346	0,01039787
MERTK	2,17534855	0,0105061
STAB1	1,42961789	0,01067038
SNHG19	-0,69253814	0,01067038
GPR34	1,71852065	0,01154955
PGK1P1	-0,89421126	0,01190472
SLAMF8	4,08148284	0,01245686
DPYSL3	-2,18447803	0,01247121
CROT	0,69221548	0,01248724
SYNGR1	-0,49112322	0,01248724
KCNG1	-1,86267072	0,01282685
ODC1	-0,4556527	0,01301026

MT-CO2	0,54761616	0,01301026
PLEKHH3	-0,69610833	0,01355716
SCN4B	1,84071947	0,01364432
CDCA7	-1,12187728	0,01414454
HYOU1	0,48453579	0,01533814
ALOX5AP	0,95708777	0,01533814
MCC	1,03224059	0,01593128
PPP1R3E	-0,56083696	0,0160832
GCAWKR	-2,51577613	0,0160832
ALDOA	-0,5560333	0,0160832
KANK2	-0,4052521	0,01676801
FGFR3	-1,11396345	0,0174561
RTL5	2,03015578	0,0174561
SLCO4A1	-1,10931698	0,0174561
HADH	-0,58519983	0,01904544
AMOTL1	-0,92226607	0,01904544
MPIG6B	1,79463089	0,01929308

Supplementary Table S3. Pipette scheme Master Mix cDNA synthesis per reaction

Reagent	Volume [µl]
5x Reaction Buffer	4
RevertAid H Minus Reverse Transcriptase	1
RiboLock RNase inhibitor	0.5
dNTP Mix	1

Supplementary Table S4. cDNA synthesis program

Step	Temperature [°C]	Time [min]
Primer annealing	25	5
Reverse transcription	42	60
Inactivation Reverse transcription	70	5

Supplementary Table S5. Pipette scheme RT-qPCR for SYBR Green Master Mix per reaction

Reagent	Volume [µl]
Maxima SYBR Green qPCR Master Mix	10
Primer forward (2 µM)	3
Primer revers (2 µM)	3
RNAse free H_2O	2
cDNA (50 ng)	2

Supplementary Table S6. Pipette scheme RT-qPCR for 18s Maxima Probe Master Mix per reaction

Reagent	Volume [µl]
Maxima Probe qPCR Master Mix	10
Primer forward (2 µM)	3
Primer revers (2 µM)	3
RNase free H_2O	1
cDNA (50 ng)	2
18S rRNA probe (4 µM)	1

Supplementary Table S7. RT-qPCR program

Step	Temperature [°C]	Time	
Initial denaturation	95	10 min	
Denaturation	95	14 sec	45 cycles
Primer annealing	60	1 min	
Melting curve	65-95	0.06 °C/1sec	
Cooling	37	5	
